# Evaluation of the Prevalence of Temporomandibular Joint Involvement in Rheumatoid Arthritis Using Research Diagnostic Criteria for Temporomandibular Disorders

**Published:** 2018-11

**Authors:** Nazanin Mortazavi, Mansour Babaei, Neda Babaee, Hamed Hossein Kazemi, Roozbeh Mortazavi, Amrollah Mostafazadeh

**Affiliations:** 1 Assistant Professor, Department of Oral and Maxillofacial Medicine, Dental School, Golestan University of Medical Sciences, Gorgan, Iran; Dental Research Center, Golestan University of Medical Sciences, Gorgan, Iran; 2 Assistant Professor, Department of Rheumatology, Medical School, Babol University of Medical Sciences, Babol, Iran; 3 Associate Professor, Department of Oral and Maxillofacial Medicine, Dental School, Babol University of Medical Sciences, Babol, Iran; 4 Assistant Professor, Department of Oral and Maxillofacial Medicine, Dental School, Babol University of Medical Sciences, Babol, Iran; 5 Assistant Professor, Department of Internal Medicine, Medical School, Shiraz University of Medical Sciences, Shiraz, Iran; 6 Associate Professor, Department of Immunology and Serology, Medical School, Babol University of Medical Sciences, Babol, Iran

**Keywords:** Rheumatoid Arthritis, Temporomandibular Joint Disorders, Anti-Cyclic Citrullinated Protein Antibodies, Rheumatoid Factor

## Abstract

**Objectives:**

Temporomandibular joint (TMJ) disorders, known as TMDs, are significant public health problems and may result in pain and disability. In order to determine the prevalence of clinical/subjective TMD in rheumatoid arthritis (RA), we used the research diagnostic criteria (RDC)/TMD axes. We assessed the anti-cyclic citrullinated protein (anti-CCP)-related TMD in RA for the first time.

**Materials and Methods:**

Fifty-two RA patients were compared to 47 healthy controls with regard to complete blood count (CBC), serology, acute phase reactants (APR), and TMJ dysfunction.

**Results:**

The anti-CCP antibody showed a significant correlation with the development of clinical TMD (P=0.001, 95% confidence interval (CI)=12.4%–35.6%). A prevalence of 50% was calculated through the RDC/TMD for such disorders. In RA patients, statistically significant differences were observed between the groups with and without clinical TMD regarding psychological depression and physical symptoms.

**Conclusions:**

According to the results, a significant correlation was found between the anti-CCP antibody and TMD. Therefore, when this antibody is detected in the blood serum, the treatment must be initiated. The RDC/TMD used in this study assessed the prevalence of TMJ dysfunction in conformity with RA-associated TMJ findings previously obtained through other conventional methods.

## INTRODUCTION

Since 2010, a positive test for the anti-cyclic citrullinated protein (anti-CCP) antibody has been included in the diagnostic criteria of rheumatoid arthritis (RA) according to the American College of Rheumatology (ACR) in partnership with the European League Against Rheumatism (EULAR) [1]. However, the clinical diagnosis of RA is primarily based on the signs and symptoms of chronic inflammatory arthritis [2]. A temporomandibular joint (TMJ) affected by RA may manifest pain, joint stiffness, changes in the jaw relation, difficulties in opening the mouth, and open bite [3,4]. In addition to the systemic inflammatory activity, the intensity of the concurrent TMJ pain can have a negative impact on daily activities and quality of life in RA patients [5]. Facial pain and jaw dysfunction constitute a large and heterogeneous group of disorders, known as temporomandibular joint disorders (TMD) [6,7]. The actual TMD prevalence has been a matter of debate due to inconsistent criteria used for its assessment. To overcome this inconsistency, the research diagnostic criteria for TMD (RDC/TMD) was introduced in 1992 [8]. To date, however, the axes (clinical and subjective) of the RDC/TMD and its latest version, i.e. the diagnostic criteria for TMD (DC/TMD) [9], have not been applied in the diagnosis of RA. Therefore, the related literature is lacking a standardized methodology for the assessment of concurrent TMD. Moreover, unlike the traditional rheumatoid factor (RF) [10], there is still no proof for the presence of a critical association between the anti-CCP antibody and TMD. The present clinical trial was therefore designed to explore the TMD prevalence in RA using the RDC/TMD. We looked for the anti-CCP-related TMD in this disabling disease for the first time.

## MATERIALS AND METHODS

This study has been approved by the Ethics Committee of Babol University of Medical Sciences (MUBABOL.REC.1392.18). All subjects gave their informed consent before participating in the study. This investigation is in accordance with the revised Helsinki Declaration (1983). During the period from September 2013 to June 2014, 52 consecutive patients (7 men and 45 women) were examined at the rheumatology clinic of Babol University of Medical Sciences. RA diagnoses were made or confirmed through the 2010 ACR/EULAR criteria [1] by an expert rheumatologist (M.B.). Likewise, 47 healthy individuals (7 men and 40 women) volunteered among the employees of the University, who had negative RA history and were not clinically diagnosed as RA patients. These volunteers were selected as healthy controls. All the patients and the controls were over 25 years of age. Individuals with a history of trauma to the TMJ, patients who were treated for their TMJ problems, and those who were diagnosed as psoriatic arthritis patients were excluded.

### The RDC/TMD:

The translations and the examiner training program for the RDC/TMD applied in the present study can be obtained in detail from the International RDCTMD Consortium Network (www.rdc-tmdinternational.org).

### Axis I - Physical diagnosis:

The physical examination of the TMJ and the adjacent musculature was performed by an oral medicine specialist (N.M.).

The clinical diagnoses in the present study were made according to the guidelines of the RDC/TMD (2011 format) for individuals with ≥1 TMD [8,11], as follows:

Group I: Myofascial Pain Syndrome (MPS; one diagnosis per subject).
Group II: Disc Displacement Disorder (one diagnosis per joint).
Group III: Degenerative Joint Disease (DJD; one diagnosis per joint) including arthralgia, osteoarthritis, and osteoarthrosis of the TMJ.

### Axis II - Psychological assessment:

A 31-item questionnaire was completed by the subjects of the case and control groups [12]. The subjective parameters, considered in the RDC/TMD (2011 format), were as follows:

- Functional limitation of the mandible expressed as discomfort during chewing, drinking, yawning, laughing, etc.
- The degree of depression.
- Characteristic pain intensity (CPI).
- Disability days and disability scores.
- Disability points (0–6), comprising disability days (0–3) and disability scores (0–3).
- Chronic pain grades, as follows:
  Grade 0: No TMD-related pain in the last 6 months.
  Grade I: Low disability/low intensity; CPI<50; < 3 disability points.
  Grade II: Low disability/high intensity; CPI≥50; < 3 disability points.
  Grade III: High disability/moderately limiting; 3 to 4 disability points.
  Grade IV: High disability/severely limiting; 5 to 6 disability points.

### Serology:

The blood samples taken from the participants of this study, including the controls and RA-diagnosed patients, were used to measure complete blood count (CBC), C-reactive protein (CRP), erythrocyte sedimentation rate (ESR), immunoglobulin M (IgM)-RF, and anti-CCP antibody (Axis-Shield^®^ Diagnostics Ltd., Dundee, Scotland, UK) which was applied as a commercial anti-CCP. The Axis Shield® anti-CCP assay is an enzyme-linked immunosorbent assay (ELISA) based on the detection of autoantibodies in human serum or plasma towards a synthetic cyclic peptide containing modified arginine residues (CCP2 peptides) [13]. Being a semi-quantitative anti-CCP, the Axis-Shield^®^ had cut-off points of≤5 U/ml (negative) and >5 U/ml (positive). The Axis-Shield^®^ has reported anti-CCP levels below 2 U/ml as the negative control concentration [13]. The selection of the Axis-Shield® anti-CCP assay and its application at the main medical laboratory in the provincial city of Babol were incidental. The methods of the application of acute phase reactants (APR) and autoantibodies were in accordance with the manufacturers’ recommendations.

### Statistical Analysis:

Independent-samples t-test was used for the comparison of the means between the groups. The association among the qualitative variables was determined using Chi-square test. The level of significance was set at 0.05. The analyses were performed using SPSS/PC software (Version 22; SPSS Inc., Chicago, IL, USA).

## RESULTS

The mean age of RA patients versus healthy controls was 52.01±13.21 years versus 53.40±11.15 years; the difference was not statistically significant (P=0.57; Independent-samples t-test). RA was found 6 times more frequently in women than in men. Statistical tests such as Fisher’s exact test and Independent-samples t-test (for equality of the means) have determined the significance of CBC, RF, anti-CCP, and positive APR in RA patients versus healthy controls (P<0.05). Except for white blood cells (WBC) and age, other rheumatological factors showed statistically significant differences compared to healthy controls (Table 1).

**Table 1.** Rheumatological factors in rheumatoid arthritis (RA)

	** Healthy controls vs **	** RA patients **	** Statistical significance(P<0.05) **
** Age (years) **	53.404±11.563	52.109±13.2154	P=0.565 (t-test for equality of the means)
** Anti-CCP (Quantity) **	0.511±1.1544	158.318±240.3920	P<0.0001(t-test for equality of the means)
** Anti-CCP (Quality) **	2.12%	94.23%	P<0.0001 (Fisher’s exact test)
** RF **	4.25%	86.53%	P<0.0001 (Fisher’s exact test)
** WBC **	7844.681±1812.7338	8480.481±2291.5001	P=0.132 (t-test for equality of the means)
** Platelets **	268.89±55.848	315.08±108.956	P=0.009 (t-test for equality of the means)
** Hemoglobin **	12.904±1.2237	12.135±1.4207	P=0.005 (t-test for equality of the means)
** CRP **	2.12%	46.15%	P<0.0001 (Fisher’s exact test)
** ESR **	42.59%	57.69%	P=0.161 (Fisher’s exact test)

Anti-CCP=Anti-Cyclic Citrullinated Protein, RF=Rheumatoid Factor, WBC=White Blood Cell, CRP=C-Reactive Protein, ESR=Erythrocyte Sedimentation Rate

45 (86.5%) out of 49 (94.2%) high-positive anti-CCP carriers showed statistically significant differences compared to 26 (50%) out of 45 (86.5%) high-positive RF individuals (P<0.001; Pearson's Chi-square test; Table 2).

**Table 2.** Distribution of rheumatoid factor (RF) and anti-cyclic citrullinated protein (Anti-CCP) antibodies in rheumatoid arthritis (RA) versus healthy controls

	** RA patients n=52 **			** Healthy controls n=47 **	

** Antibody **	** Negative N(%) **	** Positive N(%) **		** Negative N(%) **	** Positive N(%) **
		High-positive >3 times ULN	Low-positive≤ 3 times ULN		
** RF **	7(13.46)	26(50)	19(36.53)	45(95.74)	2(4.25)
** Anti-CCP **	3(5.76)	45(86.53) ^*^	4(7.69)	46(97.87)	1(2.12)

ULN=Upper Limits of Normal;

* Statistically significant, Pearson’s Chi-square test: Χ ^2^ =67.35, P<0.001, 95% confidence interval (CI)=74.25–94.55

High-positive RF or high-positive anti-CCP antibodies comprise concentrations > 3 times the upper limit of normal. Low-positive RF or low-positive anti-CCP antibodies include concentrations ≤ 3 times the upper limit of normal [1]. The calculated specificities of RF and anti-CCP antibodies were 96% and 98%, respectively. The calculated sensitivities of RF and anti-CCP antibodies were 87% and 94%, respectively. As seen in Table 3, MPS and DJD in RA were statistically significant compared to healthy controls (P<0.001; Chi-square test). Of subjective TMDs, functional limitation of the mandible and disability in RA were also statistically significant compared to healthy controls (P<0.001; Chi-square test).

**Table 3.** Temporomandibular disorders (TMD) in rheumatoid arthritis (RA) patients and healthy controls

** TMD (RDC/TMD) **		** Healthy controls n=47 **	** RA patients n=52 **	** P Value **
** Axis I **	Myofascial pain syndrome ^*^	0	18	P<0.001
** Physical **	Disc displacement disorder	3	10	P=0.1
** diagnosis **	Degenerative joint disease ^*^	0	14	P<0.001
** Total **	Concurrent clinical TMD	3	26	
** Axis II **	Pain intensity ≥ 50/100	0	6	P=0.048
** Psychological assessment **	Low disability/low intensity (I)	0	6	P=0.048
	Low disability/high intensity (II) ^**^	0	11	P<0.001
	Functional limitation of the mandible ^**^	1	16	P<0.001
** Total **	Concurrent subjective TMD	1	17	

* Statistically significant clinical TMD,

** Statistically significant subjective TMD, RDC/TMD=Research Diagnostic Criteria for Temporomandibular Disorders

Depression index and non-specific physical symptoms (pain included and pain excluded) among RA patients are shown in Table 4. Table 5 clearly demonstrates significant differences between homologous RA groups with and without TMD in their detailed psychological factors (depression index and non-specific physical symptoms).

**Table 4.** Depression index and non-specific physical symptoms in rheumatoid arthritis (RA) patients

	** Normal **	** Moderate **	** Severe **
Depression index	<1.550	1.550–2.137	> 2.137
Non-specific physical symptoms (pain included)	<1.580	1.580–2.145	> 2.145
Non-specific physical symptoms (pain excluded)	<1.500	1.500–2.000	> 2.000

**Table 5.** Depression index and non-specific physical symptoms in rheumatoid arthritis (RA) patients with and without clinical TMDs

** RA group **		** N **	** Mean **	** Std. Deviation **	** Std. Error **	** Statistical significance (P<0.05) **
Depression index	TMD	26	1.83	0.83	0.16	Independent-samples t-test (P=0.037) ^*^
	Non-TMD	26	1.35	0.77	0.15	
Non-specific physical symptoms (pain included)	TMD	26	1.93	0.83	0.16	Independent-samples t-test (P=0.001) ^*^
	Non-TMD	26	1.15	0.72	0.14	
Non-specific physical symptoms (pain excluded)	TMD	26	1.76	0.85	0.17	Independent-samples t-test (P=0.003) ^*^
	Non-TMD	26	1.06	0.74	0.14	

* One-sample Kolmogorov-Smirnov test was carried out before this test to determine the normality of the groups

Both anti-CCP and RF are related to clinical TMD (≥1 TMD). Anti-CCP has a significant correlation with TMD (P=0.0010, 95% CI=12.4%–35.6%), and RF is significantly correlated with TMD (P=0.0026, 95% CI=9.9%–32.1%). There was no missing data in our experiment.

## DISCUSSION

The sex ratio (6:1) obtained in the present study was consistent with the previously reported 2:1 or 3:1 ratio in epidemiological surveys [2].

Being included in both clinical prediction rules (CPRs; 2007) and the ACR/EULAR (2010) for disease prediction and diagnosis [14,15], a combination of RF and anti-CCP antibodies provides the highest prognostic value in the prediction of RA [16]. However, higher titers of anti-CCP are almost exclusively observed in RA patients [17]. Such differential higher titers of anti-CCP are found in RA patients more often than in healthy controls (P<0.0001; Fisher’s exact test; Table 1). Earlier, associations between rheumatological factors, such as RF and TMJ involvement in RA, have been demonstrated [10].

A direct link between the anti-CCP antibody and clinical TMD was found in the present study (P=0.001, 95% CI=12.4%–35.6%). We should keep in mind that both autoantibodies precede the onset of RA and that the anti-CCP presents with more specificity than RF. Therefore, in comparison to IgM-RF [17], the anti-CCP related to clinical TMD could signal the medical team to initiate the treatment. Moreover, a history of systemic disease and smoking could be significant contributors to RA [18].

An exclusively published article on the RDC/TMD in RA patients showed that 75% of the patients had pain in the orofacial region [19]; this was nearly identical to our rough estimate of total clinical TMD (80%; Table 3).

The results of conventional studies have indicated that more than 50% of patients with RA have exhibited a clinically evident TMJ involvement [20]. Iraqi Kurdish patients with RA have been recently characterized by a 54% frequency [21].

In another RA study in China, only half (51.8%) of complicated patients developed severe TMD [3]. Greater clinical signs of TMJ dysfunction (65%) were observed in Albanian hospitalized patients [22]. Our study’s structure and results resembled a Norwegian controlled research in the field of RA wherein 53.1% of patients had TMJ symptoms related to general disease activity [10]. The present study was similar to a case-control study performed by Helenius et al [20], which indicated moderate to severe TMJ symptoms. Similar to their subjective responses (28%), our subjective assessment of pain, disability, and limited movement was 32.7%. The clinical examination of TMJ in our study was carried out by only one examiner to avoid any kind of bias. Criticism was raised against the RDC/TMD system regarding the overrepresentation of muscle disorder diagnoses. Muscle disorders, also called myofascial pain syndrome (MPS), were the most common diagnosis (45.3%) amongst 3000 TMD patients [8] and resembled our most common clinical finding of 34.5% (Table 3). Our results indicating the statistical significance of limited mouth opening and TMJ pain (Table 3) have been previously understood [23]. Recently, DC/TMD equipped with x-ray has been intended for implementation in the clinical and research settings [9].

A superseding result in our study was that half of RA patients who had clinical TMD developed both psychological depression and nonspecific physical symptoms in comparison with the other half who did not have such disorders (Table 5). With reference to “TMD” as a pain dysfunction syndrome (PDS), Humphris and Ling [24] have called this depression a feature of PDS. Nonspecific physical symptoms (somatization) have a tendency to accompany bodily symptoms with no physical causes. Noteworthy, physiological factors and possibly mental illness should be suspected whenever conventional physical approaches are not helping RA patients [24]. It has been reported that self-applied physical therapy, including medically advised exercise, heat or cold, or reducing jaw function, affected 75% of patients positively [25].

## CONCLUSION

Anti-CCP antibody may predict not only RA but also TMJ disorders associated with the disease. The association between RA and TMJ disorders can be significant; therefore, physicians should watch for signs and symptoms of this sensitive body joint.

The clinical and subjective axes of standardized RDC/TMD system, used for the first time in an RA study, assessed the prevalence of TMJ dysfunction in conformity with the RA-associated TMJ findings previously obtained through other conventional methods. A thorough disintegration of physical and subjective disorders, however, can be an advantage of this standardized system over traditional methods.
